# Treatment of Soft Tissue Filler Complications: Expert Consensus Recommendations

**DOI:** 10.1007/s00266-017-1063-0

**Published:** 2018-01-05

**Authors:** Fernando Urdiales-Gálvez, Nuria Escoda Delgado, Vitor Figueiredo, José V. Lajo-Plaza, Mar Mira, Antonio Moreno, Francisco Ortíz-Martí, Rosa del Rio-Reyes, Nazaret Romero-Álvarez, Sofía Ruiz del Cueto, María A. Segurado, Cristina Villanueva Rebenaque

**Affiliations:** 1 Instituto Médico Miramar, Paseo de Miramar 21, 29016 Málaga, Spain; 2 Centro de Medicina Estética Dra Escoda, Rambla de Catalunya 60, Barcelona, Spain; 3 Clínica Milénio, R. Manuel da Silva Leal 11C, Lisbon, Portugal; 4 Centro Médico Lajo-Plaza, Calle Moreto 10, Madrid, Spain; 5 Clínica Mira + Cueto, Av. de Concha Espina 53, Madrid, Spain; 6 Clínica Oftalmológica Antonio Moreno, Calle Esperanto, 19, 29007 Málaga, Spain; 7 Teknobell Médicina Estética, Av. Pdte Carrero Blanco 14, Seville, Spain; 8 Grupo de Dermatología Pedro Jaén, Calle Cinca 30, Madrid, Spain; 9 Clínica de Medicina Estética Dra. Nazaret Romero, Paseo Castellana 123, Madrid, Spain; 10 SClinic, Claudio Coello 92, Madrid, Spain; 11 Hospital del Sureste Vía Verde, Ronda del Sur 10, Arganda del Rey, Madrid, Spain; 12 Clínica de Medicina Estética Dra Villanueva, Carrer de Calvet 10, Barcelona, Spain

**Keywords:** Esthetic procedures, Dermal fillers, Complications, Treatment

## Abstract

**Background:**

Dermal fillers have been increasingly used in minimally invasive facial esthetic procedures. This widespread use has led to a rise in reports of associated complications. The aim of this expert consensus report is to describe potential adverse events associated with dermal fillers and to provide guidance on their treatment and avoidance.

**Methods:**

A multidisciplinary group of experts in esthetic treatments convened to discuss the management of the complications associated with dermal fillers use. A search was performed for English, French, and Spanish language articles in MEDLINE, the Cochrane Database, and Google Scholar using the search terms “complications” OR “soft filler complications” OR “injectable complications” AND “dermal fillers” AND “Therapy”. An initial document was drafted by the Coordinating Committee, and it was reviewed and modified by the experts, until a final text was agreed upon and validated.

**Results:**

The panel addressed consensus recommendations about the classification of filler complications according to the time of onset and about the clinical management of different complications including bruising, swelling, edema, infections, lumps and bumps, skin discoloration, and biofilm formation. Special attention was paid to vascular compromise and retinal artery occlusion.

**Conclusions:**

Clinicians should be fully aware of the signs and symptoms related to complications and be prepared to confidently treat them. Establishing action protocols for emergencies, with agents readily available in the office, would reduce the severity of adverse outcomes associated with injection of hyaluronic acid fillers in the cosmetic setting. This document seeks to lay down a set of recommendations and to identify key issues that may be useful for clinicians who are starting to use dermal fillers. Additionally, this document provides a better understanding about the diagnoses and management of complications if they do occur.

**Level of Evidence V:**

This journal requires that authors assign a level of evidence to each article. For a full description of these Evidence-Based Medicine ratings, please refer to the Table of Contents or the online Instructions to Authors www.springer.com/00266.

## Introduction

Dermal fillers have been injected with increasing frequency over the past three decades for soft tissue augmentation by volume expansion in the management of the aging face. Over the past several years, the number of procedures involving soft tissue fillers has increased from 1.6 million per year in 2011 to more than 2.4 million in 2015 [1].

The growing use of dermal fillers, specifically the use of hyaluronic acid (HA), can be explained by their effectiveness and versatility as well as their favorable safety profiles.

Although the incidence of complications is low and the majority of adverse events are mild, the increase in the number of procedures has produced the concurrent increase in the number of complications [2–4]. Among these, serious occurrences are fortunately rare, although probably underreported.

It is noteworthy that proper selection and placement of product can help avoid some complications [5].

The classification of filler complications can be divided according to severity (mild, moderate, or severe); nature (ischemic complications and non-ischemic); or by the time of the onset (early or late) [6, 7]. A classical classification proposed by Rohrich et al. [8] suggested that complications should be classified as early, late, and delayed, roughly defined as less than 14, 14 days to 1 year, and more than 1 year, respectively, as these time frames correlate well with the potential underlying etiology. Although the panel proposes to classify filler complications as immediate onset (up to 24 h after procedure); early onset (24 h to 4 weeks); and delayed onset (more than 4 weeks), to facilitate the understanding and follow-up of the manuscript, the immediate- and the early-onset complications have been listed together.

Although different papers about the management of dermal filler complications have emerged in the last years [2–4, 6, 7, 9–13], optimal complication management remains an unmet need in the field of esthetic medicine.

This paper aims to describe potential adverse events associated with dermal fillers and to provide guidance on their treatment and avoidance.

## Methods

On November 2016, a multidisciplinary group of experts in esthetic treatments, selected based on their level of expertise in this subject, convened to discuss the management of the complications associated with dermal fillers use. Among the different topics discussed in the meeting, the classification of the filler complications and the management of such complications have emerged as key issues. The authors developed this consensus paper based on those discussions and a review of the current literature.

Searches of MEDLINE (from 2000 to November 2016), the Cochrane Database (from 2000 to November 2016), and Google Scholar were conducted using the search terms “complications” OR “soft filler complications” OR “injectable complications” AND “dermal fillers” AND “Therapy”. References cited in selected articles were also reviewed to identify additional relevant reports. Limits were set for articles written in English, French, and Spanish with human subjects. Additional data were identified through bibliographic reviews. Additionally, relevant published national and international guidelines were also scrutinized.

Because of the nature of esthetic procedures, which are usually elective processes, it is not easy to devise meaningful prospective clinical trials that evaluate complications. There are a few prospective trials, but these are often not randomized or controlled. Therefore, our knowledge base mainly comprised case reports and summaries of individual practitioner’s experience.

**Fig. 1:**
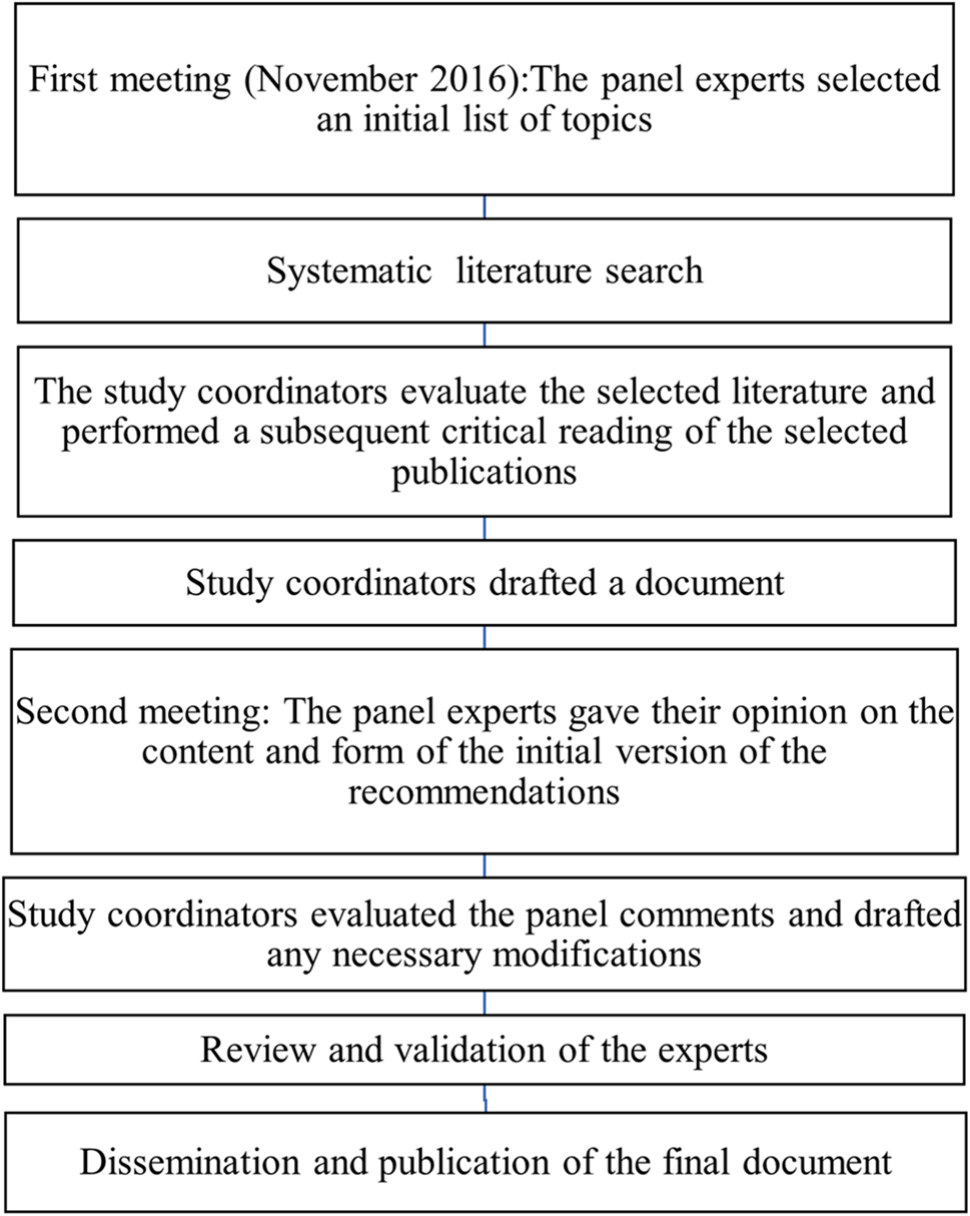
Flow diagram of the consensus process

An initial document was drafted by the Coordinating Committee, and it was reviewed by the expert panel members. The Coordinating Committee evaluated the panel’s comments and modified the draft as they considered necessary. Subsequent revisions were based on feedback from the other authors until a consensus was achieved, and the final text was then validated (Fig. 1).

The recommendations expounded in this document represent the panel’s expert opinion based on their clinical experience as well as on published data regarding dermal filler complications in esthetic procedures.

## Results

According to the time of onset, the panel proposes to classify filler complications as immediate onset (up to 24 h after procedure); early onset (24 h to 4 weeks); and delayed onset (more than 4 weeks). Nevertheless, to make the manuscript reading more pleasant, the immediate- and the early-onset complications have been listed together. The main types of adverse events by time of onset are illustrated in Table 1.

**Table 1:** Overview of the adverse events associated with the use of dermal fillers. Adapted from Funt and Pavicic [6]

Adverse events	Signs and symptoms	
	Immediate/early adverse events^b^	Delayed adverse events^c^
Injection site reactions^a^	Erythema Edema Pain/tenderness Bruising Itching	Erythema Edema Pain/tenderness Nodule/abscess Systemic responses Biofilm
Infection	Erythema Edema Pain/tenderness Acne papule formation Nodule/abscess Herpes outbreak	Biofilm Herpes outbreak Foreign-body granuloma^d^
Hypersensitivity	Erythema Edema Pain/tenderness Non-fluctuant nodules	Migration of filler material
Technical and placement errors	Bumps/lumps Asymmetries Contour irregularities Compromised muscle function Dysesthesias, paresthesias, and anesthesia	Immune reactions Compromised muscle function Dysesthesias and paresthesias
Skin discoloration	Redness Whiteness Hyperpigmentation	Persistent discoloration Persistent scarring
Vascular compromise^e^	Blurred vision Loss of vision Pain Blanching	Tissue necrosis

^a^Atypical as a delayed adverse events

^b^Occurring up to several days post-treatment

^c^Occurring from weeks to years post-treatment

^d^Varying from subclinical histologic changes to disfiguring nodules

^e^Retinal artery occlusion

### Immediate- and Early-Onset Dermal Filler Complications

#### Bruising/Ecchymosis

Bruising is an understandable and common complication, though unwelcome by patients, of filler injections. Bruising is observed more frequently after injection into the dermal and immediate subdermal planes using fanning and threading techniques [14].

Bruising may be treated with cold compresses after the procedure, arnica, aloe vera, or vitamin K creams [6, 15, 16]. The risk of bruising may be reduced by injecting the filler slowly. If bruising appears, it can be reduced by pressing with a compress [2].

Different substances associated with anticoagulation including nonsteroidal anti-inflammatory drug medications, many vitamin/herbal supplements, and antiplatelet should be discontinued 7–10 days (not without consultation with the treating physician) prior to treatment to reduce the risk of bruising [2, 16, 17].

Clinical studies evaluating the risk of bleeding in patients undergoing minor dental surgery procedures have reported conflicting results. Several studies reported that the postoperative bleeding rate in patients undergoing oral anticoagulant treatment, such as warfarin or coumadin, was not higher than that in patients not undergoing oral anticoagulant treatment [18–20]. However, some studies have reported more postoperative bleeding in oral anticoagulant treated patients [21, 22]. The results of a recently published meta-analysis found that although patients treated with oral anticoagulants have a higher postoperative bleeding risk than those not treated with oral anticoagulants following minor dental surgery, local hemostatic methods effectively stopped the bleeding [23].

An expert group consensus report, focused on preventing dermal fillers complications, recommended to reduce the risk of bruising and to pay special attention in patients taking oral anticoagulants [24].

In summary, the risk of bleeding in patients taking oral anticoagulant treatments and with a stable international normalized ratio (INR) in the therapeutic range 2–4 is really small, and its discontinuation may increase the risk of thrombosis [25].

According to the panel opinion, if the anticoagulant treatment is well balanced, the associated risk of discontinuing the treatment is greater than that of bleeding.

Although it is not forbidden, it is advisable to avoid strenuous exercise for 24 h to reduce the risk of bruising and swelling [17].

Regarding this issue, the panel recommends:

- Prophylaxis: To use arnica with vitamin K creams for 3 to 4 days.
- Treatment: To use arnica and vitamin K creams/photoprotection.

#### Swelling and Edema

Some transient swelling in the immediate postprocedural period is normal and occurs with all dermal fillers, but may vary in timing and severity depending on the specific product used [6, 16]. Besides injection volume and technique, patient factors, such as dermographism, may also influence the amount of swelling. The most commonly affected areas are the lips and the periorbital region. In patients with a long-lasting lip augmentation procedure a transient swelling of the lips may occur.

It should be mentioned that this swelling should not be confused with an antibody-mediated edema (angioedema), which is extremely rare [19, 26].

The panel recommends:

- Prophylaxis:
  i. Anti-inflammatory Enzyme:
    1. Wobenzym Vital^®^ (Diafarm, 08210 Barberà del Vallès, Barcelona, Spain): 2 capsules/12 h.
    2. Bromelain: 300 mg/day, divided in three doses.
  ii. Arnica/gelsenium: 4–5 pills/24 h 3–4 days.
  iii. Cold compresses (about 5 min).
- Treatment:
  i. Mild: Cold compresses/anti-inflammatory enzyme (Wobenzym Vital^®^ or Bromelain, according to the previous dosage)/observation.
  ii. Moderate:
    1. Streptokinase/streptodornase (10,000/2500 U): 2 pills/8 h, for 3–6 days.
    2. Nonsteroidal anti-inflammatory drugs (NSAIDs):
      Cox 1: Ibuprofen 400–600 mg/8 h.
      Cox 2: Celecoxib (200–400 mg/24 h).

The different NSAIDs and anti-inflammatory enzyme treatments are summarized in Table 2.

**Table 2:** Different NSAIDs and anti-inflammatory enzyme treatments recommended by the panel

Product	Dose	Comment
Diclofenac 50 mg	1/12 h	Associated with some gastric protector (no more than 5 days)
Varidase	4–8 pills/6 H and after 2 pills/8 h	For 7–10 days and after for 3–6 days
Bromelin 50 mg^a^	4–8 pills/24 h	For 3–6 days
Bromelin, papain, trypsin, and quimotrypsin^b^	4–8 pills/24 h	For 3–6 days
Ibuprofen	400–600 mg/8 h	For 2–3 days
Dexketoprofen trometamol	25 mg/8 h	For 1–3 days
Acetyl salicylic acid	100 mg/24 h	For 7 days (if necrosis)

^a^Fortilase^®^, MEDA PHARMA SL, Avenida de Castilla, 2. San Fernando de Henares, Madrid. Spain

^b^Wowenzym Vital^®^; Diafarma Laboratories, 08210 Barberà del Vallès, Bacrelona. Spain

Before prescribing NSAIDs, it is important to consider the following recommendations:

- To use the lowest dose and for the shortest time.
- To select the NSAID according to the drug profile and the patient risk factors.
- To use gastroprotective agents for minimizing the gastrointestinal harm associated with use of NSAIDs.
  iii. Severe:
    1. Prednisone: 1 mg/kg/day + pantoprazole 40 mg. Approximately for 3 days (according to the clinical course).
    2. Deflazacort: 1–1.5 mg/kg/day + pantoprazole 40 mg. Approximately for 3 days (according to the clinical course).
    3. In case of necrosis: lymphatic drainage and soft massage.

The different steroid treatments are summarized in Table 3.

**Table 3:** Different steroid treatments recommended by the panel

Product	Dose	Comment
Deflazacort^a^	1–1.5 mg/kg/day	For 15–21 days. Associated to some gastric protector
Prednisone 30 mg	1 pill/24 h	For 3 days
	30–60 mg/24 h	For 2–3 weeks (corticoids in decreasing doses)
Methylprednisolone	40–80 mg/24 h	For 2–3 weeks (corticoids in decreasing doses)

^a^Deflazacort is the first-line treatment. The length of the treatment should be from 3 to 6 weeks, prescribing the drug at increasing doses each week; i.e., first week 0.5 mg/kg/day until reaching 1.5 mg/kg/day. Subsequently, corticoids in decreasing doses

Because dermal fillers are essentially foreign bodies, some patients may develop hypersensitivity to injected products due to an immunoglobulin E (IgE)-mediated immune response (Type I hypersensitivity reaction). Angioedema occurs within hours of exposure, although the reactions can be severe and can last for several weeks [27].

This angioedema will usually subside within a few days with antihistamines and/or oral steroids. The patient should be closely monitored to rule out possible infection.

Additionally, delayed hypersensitivity reactions, which typically occur 1 day after injection, are characterized by induration, erythema, and edema, and are mediated by T lymphocytes rather than antibodies [28]. Delayed hypersensitivity reactions are non-responsive to antihistamines. In the case of HA, this will involve treatment with hyaluronidase.

#### Erythema

Immediately after injection, some skin redness may occur and is normal. Treatments for rosacea may be effective, including oral tetracycline or isotretinoin [6]. A medium-strength topical steroid is advocated for persistent erythema. However, long-term use of high-potency steroids should be avoided. Additionally, vitamin K cream may be useful in accelerating resolution of erythema [29].

#### Infections

Any procedure that breaks the surface of the skin carries with it a risk of infection, and injecting dermal fillers is no exception. Acute infections, which appear as acute inflammation or abscesses at the site of injection, are typically due to common pathogens present on the skin such as *Staphylococcus aureus* or *Streptococcus pyogenes*.

If untreated, the conditions may lead to sepsis, particularly in elderly people or in patients with other conditions that alter the immune system. Mild forms may be treated with oral antibiotics, while more serious ones require intravenous antibiotics and hospitalization [6, 30].

The panel recommends:

i. Amoxicillin clavulanic acid 4 g/24 h 15 days.
ii. Ciprofloxacin 500–750 mg bid for 2–4 weeks.

The different antibiotics recommended by the panel are listed in Table 4.

**Table 4:** Different antibiotic treatments recommended by the panel

Product	Dose	Comment
Amoxycillin/clavulanic acid	4 g/24 h	For 10–15 days
Cloxacillin	3 g/24 h	For 10–15 days
	500 mg/8 h	For 30 days
Ciprofloxacin	500 mg/8 h	For 3–6 weeks
Azithromycin	500 mg/24 h	For 3 days
Minocycline	500 mg/12 h	For 30 days
Flucloxacillin	500 mg/8 h	For 7 days

#### Herpetic Outbreak

Dermal filler injections can lead to reactivation of herpes virus infections. The majority of herpetic recurrences occur in the perioral area, nasal mucosa, and mucosa of the hard palate [2, 6, 16, 17].

Patients with a history of severe cold sores (more than 3 episodes) should be prescribed antiherpes medication prophylactically before treatment when injections in vulnerable areas are planned. In these patients, the panel recommends: Valaciclovir 1 g/24 h 1 day before and 3 days after filler injection.

Additionally, according to the panel recommendations, in patients with active herpes lesions, injections should be delayed until their complete resolution.

#### Dysesthesias, Paresthesia, and Anesthesia

Nerve damage during an esthetic procedure, although very rare, can occur as result of different causes such as direct trauma, injection of filler into the nerve, tissue compression by the product. Nerve injury may be either transient and reversible, or permanent. The most common site of dysesthesias, paresthesia, and anesthesia is the infraorbital nerve. Less commonly, a transient Bell’s palsy or marginal mandibular nerve dysfunction has been seen and may last several weeks [2, 6, 16, 17].

Although 71% of patients with Bell’s palsy experience complete spontaneous resolution, the remaining 29% exhibit lifelong residual hemifacial weakness. Besides the protective strategies of the ocular surface (artificial tears, occlusion, etc.), the mainstay of acute management of Bell’s palsy is a short course of high-dose (for example, 1 mg/kg) oral steroids [31]. Surgical decompression of the meatal segment, antiviral therapy, electrotherapy, physical therapy, and acupuncture has been proposed, though the evidence does not support their use [31].

The panel considers it crucial to have a thorough knowledge of facial anatomy to minimize the incidence of such complications.

#### Lumps and Bumps

Lumps and bumps are one of the most common complications associated with filler injections [16]. They can be classified according their type (non-inflammatory, inflammatory, or infectious) as well as their time to presentation (early, late, or delayed) [8]. As they can arise from a number of causes, investigation may be required to establish a diagnosis. As a general rule, early lumps and bumps present within days or weeks, tend to be painless, and are most likely the result of suboptimal techniques such as excess filler use, superficial placement, and incorrect product for the indication [16, 32]. Lumps occurring in the early post-treatment period may respond to massage.

The panel recommends:

- Observation: do not treat if the inflammation is improving.
- If the non-inflammatory lump persists, treat the over correction:
  i. Needle aspiration or minimal stab wound incision with evacuation.
  ii. Hyaluronidase 150U/mL (be aware of possible allergic reactions).
  iii. To treat the post-inflammatory hyperpigmentation: intense pulsed light/laser; photo-protection; or depigment cream.

Hyaluronidase preparation, dilution, and doses are summarized in Table 5.

**Table 5:** Hyaluronidase preparation, dilution, and doses recommended by the panel

Dilution	Dose
150 IU/mL saline	150 IU/mL
1 × 10^4^ µg in 3 mL (saline)	0.3–0.5 mL per injected point
1 × 10^3^ IU in 2–4 mL (saline)	50–200 IU in nodules
1.5 × 10^3^ IU in 10 mL (saline)	500–1.000 IU in patients at risk of necrosis
	100–200 IU 3–4 mm in depth^a^

^a^This strategy refers to the injection of hyaluronidase throughout the area around the vascular occlusion point to promote its intravascular penetration and facilitate removal of the HA that is obstructing the vessel

#### Vascular Compromise

Vascular compromise after a soft tissue filler injection is a major, immediate complication that is almost always the result of intravascular injection into an artery, causing an embolism that impedes blood flow.

The incidence of intravascular injection seems to be more frequent than we assumed. The results of an internet-based survey conducted on 52 experienced injectors worldwide showed that a 62% of them reported one or more intravascular injections [32]. Recognition of a vascular event and swift and aggressive treatment is necessary to avoid potentially irreversible complications [33–36].

The two primary diagnostic symptoms of vascular occlusion are pain and changes in skin color. Arterial occlusion is typified by immediate, severe, and disproportionate pain and color changes (white spots) [32], whereas venous occlusion may be associated with less severe, dull, or delayed pain (in some cases there may be no pain).

Because intravascular occlusions are rare events, present recommendations for prevention and management are based almost exclusively on expert opinion [37] and consensus reports [6, 16, 17, 38].

Nevertheless, when vascular occlusion is suspected, it is crucial that the injection is stopped immediately and treatment is rapidly instigated. The objective is to facilitate blood flow to the affected area. Treatment strategies include hyaluronidase, warm compress, massaging or tapping the area, and applying 2% nitroglycerin paste to promote vasodilatation [32, 39, 40].

Hyaluronidase should be injected immediately, regardless of the filler used, and administered daily in liberal doses where signs and symptoms are present [6, 16, 17, 38].

For intravascular infarction, high doses of hyaluronidase (200–300 U) have been recommended, [16, 17, 38]. When injecting hyaluronidase to treat acute ischemia, consensus recommendations are that the entire ischemic area be treated, not just the site where HA was originally injected [16, 17, 38]. If there is no improvement, the procedure should be repeated hourly until clinical resolution is achieved [16]. Doses up to 1500 U may be required to achieve reversal of vascular compromise [16, 17, 38].

The risk of an intravascular injection can be reduced by different strategies, which are listed in Table 6.

**Table 6:** Strategies for reducing the risk of skin necrosis with hyaluronic acid fillers

Panel recommendations
a. Aspirating prior to injection
b. Utilizing lower volumes and serial injections in high-risk areas
c. Treating one side at a time
d. Pinching/tenting the skin to provide more space superficial to the branches of the main arteries
e. Manual occlusion of the origin of the supratrochlear vessels with the non-dominant finger
f. Blunt cannulas may reduce, but not eliminate, the risk

### Retinal Artery Occlusion

The occlusion of the central retinal artery (CRA), or some of its branches, is a rare but devastating visual complication that can occur after an esthetic procedure with soft tissue fillers, such as autologous fat, hyaluronic acid, or collagen [41].

A literature review published in 2015 reported 98 cases of vision changes following filler injection [42]. The injection sites identified with higher risk of complications were the glabella (38.8%), nasal region (25.5%), nasolabial fold (13.3%), and forehead (12.2%) [42]. As regards, the filler type, autologous fat, was the most common causative material (47.9%) followed by hyaluronic acid (23.5%) [42].

The underlying mechanism of action leading to vision loss is retrograde flow [42, 43]. If the tip of the needle penetrates the wall of a distal branch of ophthalmic artery, the force of injection can expand arterioles and cause retrograde flow [42, 43]. Although this is the prevailing theory regarding the mechanism of occlusion, theories related to compression of vessels may also contribute [43].

The main symptom is blindness in the affected eye, usually painless, which can occur within seconds after injection. Other associated symptoms are pain at the injection site and headache [41–44].

If visual loss has occurred, therapeutic measures should be immediately implemented, because maintained CRA occlusion for more than 60–90 min causes irreversible blindness [45].

The therapeutic measures that have to be performed at the center where the procedure was made would be:

- Medical treatment [43]:
  - One drop of topical timolol 0.5% and/or an acetazolamide 500 mg tablet (after excluding allergy to sulfonamides).
  - To administer a sublingual pill (325 mg) of acetylsalicylic acid or one of nitroglycerin 0.6 mg.
  - To administer an intravenous infusion, 100 mL over 30 min, of mannitol 20%.
- Digital massage [43]: It should start immediately while preparing the treatment and to continue once the drugs have been administered.
  - The patient should be placed in a supine position.
  - Ensure the patient’s eyes are closed.
  - Apply firm pressure (enough to ensure that the eyeball is indented about 2–3 mm) on the eyeball through the closed eyelids.
  - Apply firm pressure for 5–15 s and quickly release.
  - Repeat this cycle for at least 5 min.

If despite these measures the patient does not recover the vision in the first 15–20 min, the patient must be referred to an ophthalmology-specialized center for performing an anterior chamber paracentesis for decreasing intraocular pressure [43].

Because, up to now, fibrinolytic or hyaluronidase infiltrations have not demonstrated an unequivocal efficacy; their use is not widespread [43].

Due to the seriousness of the complication, prevention through a good understanding of facial vasculature anatomy and injection techniques is extremely important.

### Late-/Delayed-Onset Dermal Filler Complications

#### Bruising

Although bruising is usually an early-onset complication, persisting staining may arise. Larger or cosmetically distressing purpura can be treated with vascular lasers, either pulsed dye light or potassium titanyl phosphate lasers, to speed recovery [6, 16].

#### Edema

##### Angioedema

Angioedema typically has an early onset; however, episodes that last more than 6 weeks may be observed. These cases are often difficult to treat and have a variable response to medication. The therapeutic approach is stepped, moving to the next step if an inadequate response was obtained. Edema should be controlled with the smallest dose of oral steroids that is effective [6]. Additional treatment options including topical or intralesional steroids, or immunosuppressive agents, have been proposed [2].

##### Non-Antibody-Mediated (Delayed) Edema

Delayed hypersensitivity reactions, which are characterized by induration, erythema, and edema, usually occur 1 day after injection, but may be seen as late as several weeks after injection and may persist for many months [46].

Antihistamines are not effective in these reactions. The best approach is to remove the allergen. If HA had been used, treatment with hyaluronidase would be recommended. Other fillers may require treatment with steroids until the filler resorbs, laser treatment, and/or extrusion [47]. Sometimes, it is even necessary to make, as a last resort, an excision.

##### Malar Edema

Malar edema is a particularly serious complication that has been frequently reported with all fillers when injected into the infraorbital hollow and tear troughs [48].

The phenomenon of malar edema can be explained by an understanding of the anatomy of the lower eyelid. Injection of fillers may cause edema by either augmenting the impermeable barrier of the malar septum (impeding lymphatic drainage) or bydirect pressure on the lymphatics when injection volumes are too large [48].

It is worth mentioning that malar edema is long lasting and responds poorly to treatment. The therapeutic strategies include head elevation, cold compresses, manual compression multiple times daily, lymphatic drainage, and methylprednisolone. In those patients treated with HA, hyaluronidase treatment should be given [48].

Nevertheless, the best approach is to reduce its incidence by patient and filler selection; limiting filler volume; and by placing filler material deep into the malar septum at the immediate pre-periosteal level [48].

Persistent periorbital edema can be observed when injecting too much volume in the tear trough or when the product is placed too superiorly and too superficially. This complication is more frequent in patients with preexisting malar edema, because the obstruction of lymphatic drainage may be an inciting factor [16].

#### Skin Discoloration

##### Neovascularization

The tissue trauma caused, as a result of tissue expansion and/or by excessive molding and massage of the filler, can favor the appearance of new capillaries, arterioles, and venules. Neovessels may appear days or weeks after the procedure, but should fade within 3–12 months without further treatment. Laser treatment has shown to be effective in these cases.

##### Hyperpigmentation

Hyperpigmentation is not an uncommon complication in dermal filler procedures, especially in subjects with Fitzpatrick skin types IV–VI, although post-injection hyperpigmentation can also be seen in other skin types [49, 50].

For managing this problem, the first therapeutic approach should be with a bleaching agent such as topical hydroquinone (2–8%) and Retin-A (tretinoin) combined with daily full-spectrum sunscreen application [6]. In those cases of resistant post-inflammatory hyperpigmentation, chemical peels may also be used. If the treatment is not successful, the next steps include the treatment with intense pulsed light, a pulsed dye laser, or fractional laser [6].

##### Tyndall Effect

When particulate HA fillers are inappropriately implanted into the superficial dermis or epidermis they cause a bluish hue referred to as “Raleigh scattering” or the “Tyndall effect” [51]. If not treated, superficial product has been commonly observed to last for very long periods of time, even years [16].

Hyaluronidase should be the initial approach to treatment. For those patients who do not achieve a good response, dyspigmentation can be treated by nicking the skin with a small-gauge needle or surgical scalpel and expressing the superficial, unwanted dermal filler [52, 53]. This therapeutic strategy may be applied immediately, or as long as 12 months or more after injection [53].

#### Infection

Delayed-onset chronic infections, which generally develop 2 or more weeks after injection, tend to affect a more generalized area and may involve an atypical organism (such as *Mycobacteria* or *Escherichia coli*). These are challenging for both diagnosis and treatment and can cause a chronic inflammatory response.

In the opinion of the panel, a sequence of treatment options similar to that in early acute infection should be followed:

- To perform a bacterial culture and clinical assessment to decide type of infection and treat with antibiotics or corticosteroids.
- It is important to do a differential diagnosis with hypersensitivity, as the use of steroids should be avoided in infection.
- There are not comparative studies supporting the effectiveness of a specific therapeutic regimen. Once the species is identified, an antibiogram is required. If atypical mycobacteria are suspected, while waiting for the antibiogram results, an empirical treatment with antibiotics, which cover atypical mycobacteria, such as claritromicina 500 mg/twice daily combined with ethambutol or rifampicin, may be recommended.

##### Abscess

Abscess formation is a rare complication, reported in permanent hydrogel fillers, occurring any time from 1 week to several years after treatment; it may persist for weeks, and periodically recur for months.

The first-line therapy is drainage and antibiotics. As mentioned for the delayed-onset chronic infections, the panel recommends, in order to tailor the treatment, to obtain bacterial cultures and perform sensitivity reports [54]. Although it is extremely rare, midfacial and periorbital infection may result in intracerebral complications [6].

It has been proposed that low-grade infections are responsible for all delayed-onset complications, including foreign-body granulomas, as a result of biofilm formation [55].

#### Nodules

Nodules and lumps are common complications resulting from the use of dermal fillers.

Nodules must be categorized as inflammatory or non-inflammatory.

##### Inflammatory Nodules

Delayed-onset nodules (from 4 weeks to 1 year or even longer) are usually inflammatory (immune responses to the filler material) and/or infection related (including biofilm) [56, 57].

Biofilms are widespread in nature and consist of densely packed communities of bacteria that surround themselves with secreted polymers. However, in patients presenting delayed inflammatory complications due to permanent filler, biofilm gained much interest after demonstrating that bacteria could be detected in biopsies, although a culture test had frequently been negative [58]. It is therefore important to use molecular techniques, such as polymerase chain reaction or fluorescence in situ hybridization tests for delayed-onset nodule complications where biofilm involvement is suspected [56].

It may be extremely difficult to distinguish inflammation due to a bacterial biofilm from a low-grade hypersensitivity reaction.

Many bacterial species form biofilms, and as biofilms progress, they become more antibiotic and culture resistant. As regards treatment, although these infections are difficult to treat, the cure is removal of the implant, which is not always possible. In those cases of HA fillers, hyaluronidase can be used. However, we must be extremely cautious because, according to the labeling, hyaluronidase should not be used in the presence of an active infection (cellulitis) as it may facilitate the spread of infection into adjacent tissues [37].

Other strategies for treating biofilm include low doses of triamcinolone mixed with 5-fluorouracil (FU) (0.1 mL triamcinolone 40 mg/mL and 0.9 mL 5-FU 50 mg/mL) injected at regular (weekly × 2, once every 2 weeks × 2, then monthly) intervals until resolution is achieved [16]. Although the reason for the therapeutic success of 5-FU remains unknown, it has been suggested that it interacts with AriR, a regulatory gene that inhibits the formation of biofilm [59].

Additionally, we have evidence supporting the use of human platelet-rich plasma in the area of the biofilm infection, with a triple effect intention: antimicrobial; for favoring HA degradation of the inflamed tissues; and to destroy the biofilm [60–62].

Regarding antibiotic treatment, a consensus report on prevention and management of HA complications recommended the following empiric antibiotic scheme: clarithromycin 500 mg plus moxifloxacin 400 mg twice daily for 10 days, or ciprofloxacin 500–750 mg twice daily for 2–4 weeks, or minocycline 100 mg once daily for 6 months [17].

##### Foreign-Body Granulomas

Foreign-body granulomas may form as the body’s immune system responds to a foreign body that cannot be broken down by the usual mechanisms.

Although they can occur with all injectable dermal fillers, the incidence is very rare (from 0.01 to 1.0%) and usually appears after a latent period, which can be several months to years after injection [63, 64]. Diagnosis of granulomas is further complicated by the fact that clinicians are sometimes faced with patients with unknown or incomplete medical and cosmetic treatment history.

Granulomatous reactions to hyaluronic acid fillers can be treated with hyaluronidase with the dosing of 150 U/mL.

Once infection has been ruled out or quiescent, granulomas may respond to oral or intralesional steroids. If steroids are not enough, many patients will respond to the addition of 5-FU to the corticosteroids. In cases of repeated failure of other therapies, surgical excision is the treatment of choice for foreign-body granuloma [2, 6, 16, 17].

#### Tissue Necrosis

Impending tissue necrosis, although fortunately rare, may occur as a result of inadvertent injection of filler into vessels supplying the mucosa or the skin, resulting in vessel occlusion. On the other hand, necrosis may also occur secondary to local edema or to occlusion of adjacent vasculature secondary to the hydrophilic properties of the product [65, 66].

The risk of skin necrosis can be reduced by different strategies (see Table 6).

All the injectors have to be familiar with the signs of skin necrosis and the appropriate therapy. For intravascular infarction, the panel recommended:

- To apply a warm gauze, tapping the area to facilitate vasodilatation, and massage of the area.
- To use topical nitroglycerin (1 or 2%) paste 2 or 3 times/daily in the office and at home by the patient. Nitroglycerin sublingual tablets can be used.
- Hyaluronidase injection (200–400 IU/1–2 mL) + massage. See Table 5.
- Although it was not absolutely proved, it was stated that acetylsalicylic acid (500 mg/8 h, 24–48 h) might be helpful.
- If there are ocular symptoms (blurred vision, loss of vision, or ocular pain), the patient has to be urgently referred to the ophthalmologist.
- Other strategies including systemic or topical steroids (prednisone 20–40 mg each day for 3–5 days), low molecular weight heparin, hyperbaric oxygen, sildenafil (1 per day for 3–5 days) have been proposed [17, 67, 68].

## Conclusions

Because of their efficacy and safety, esthetic procedures with dermal fillers have become increasingly popular. However, although the incidence of complications is relatively low and the majority of adverse events are mild, the increase in the number of procedures has been accompanied by a concurrent increase in the number of complications. As optimal complication management remains an unmet need in the field of esthetic medicine, minimizing their incidence by means of appropriate patient, product, and injection technique selection, as well as a sound understanding of facial anatomy, is probably the best approach.

Clinicians should be fully aware of the signs and symptoms related to complications and be prepared to confidently treat them. Establishing action protocols for emergencies, with agents readily available in the office, would reduce the severity of adverse outcomes associated with injection of hyaluronic acid fillers in the cosmetic setting.

It is our hope that this article will help clinicians, who are just starting to use dermal filler procedures, to effectively manage their potential complications.
